# The osteoblastic differentiation ability of human dedifferentiated fat cells is higher than that of adipose stem cells from the buccal fat pad

**DOI:** 10.1007/s00784-013-1166-1

**Published:** 2013-12-21

**Authors:** Naotaka Kishimoto, Yoshihiro Momota, Yoshiya Hashimoto, Shinichi Tatsumi, Kayoko Ando, Takeshi Omasa, Junichiro Kotani

**Affiliations:** 1 Department of Anesthesiology, Osaka Dental University, 1-5-17 Otemae, Chuo-ku, Osaka, 540-0008 Japan; 2 Department of Biomaterials, Osaka Dental University, 8-1 Hanazonocho, Kuzuha, Hirakata, 573-1121 Japan; 3 Institute of Technology and Science, The University of Tokushima, 2-1 Minamijosanjima-cho, Tokushima, 770-8506 Japan; 4 Merck Millipore, Merck Ltd., Arco Tower 5F, 1-8-1 Shimomeguro, Meguro-ku, Tokyo, 153-8927 Japan

**Keywords:** Dedifferentiated fat cells, Adipose stem cells, Ceiling culture, Buccal fat pad, Osteoblastic differentiation, Bone tissue engineering

## Abstract

**Objectives:**

The purpose of this study was to evaluate and compare the osteoblastic differentiation ability of dedifferentiated fat (DFAT) cells and adipose stem cells (ASCs) from the buccal fat pad (BFP).

**Materials and methods:**

We isolated human DFAT cells and ASCs from the BFP of a patient who underwent oral and maxillofacial surgery and then analyzed their cell surface antigens by flow cytometry. Then, the cells were cultured in osteogenic medium for 14 days. Measurement of bone-specific alkaline phosphatase (BAP), osteocalcin (OCN), and calcium deposition and alizarin red staining were performed to evaluate the osteoblastic differentiation ability of both cell types.

**Results:**

ASCs and DFAT cells were positive for CD90 and CD105 and negative for CD11b, CD34, and CD45. BAP (days 3 and 7), OCN (day 14), and calcium deposition (days 7 and 14) within DFAT cell cultures were significantly higher than those in ASC cultures. The alizarin red-stained area in DFAT cell cultures, which indicates mineralized matrix deposition, was stained more strongly than that in ASC cultures.

**Conclusions:**

The cell surface antigens of ASCs and DFAT cells tend to be similar. Furthermore, the osteoblastic differentiation ability of human DFAT cells is higher than that of ASCs from the BFP.

**Clinical relevance:**

Isolation of DFAT cells from the BFP has an esthetic advantage because the BFP can be obtained via the oral cavity without injury to the external body surface. Therefore, we consider that DFAT cells from the BFP are an ideal cell source for bone tissue engineering.

## Introduction

Adipose stem cells (ASCs) are isolated from adipose tissue and can differentiate along multiple lineages including adipocytes, osteoblasts, chondrocytes, myocytes, neuronal cells, endothelial cells, and hepatocytes [1–3]. In addition, ASCs are readily available in large quantities with minimal morbidity and discomfort associated with harvest [1–4].

ASCs are extensively used for bone tissue engineering, and their utility has been reported in various studies [5–7]. Recently, Farre-Guasch et al. [4] showed that ASCs from the buccal fat pad (BFP) can differentiate into chondrocytes, osteoblasts, and adipocytes in vitro. Shiraishi et al. [8] reported that ASCs from the BFP can form an engineered bone in the back subcutaneous pockets of nude mice. Therefore, the BFP might be a potential cell source for bone tissue engineering in oral and maxillofacial areas, because it is easy to harvest and provides a reliable volume of tissue for oral surgeons.

However, ASCs are a heterogeneous cell population, particularly at early passages, because they are obtained from the non-adipocyte fraction in adipose tissue, known as the stromal vascular fraction (SVF) [1, 3, 9]. ASCs at passage 0 include contaminating endothelial and smooth muscle cells and pericytes [1]. Therefore, other stem cell sources with a high purity are needed for improved safety in clinical application.

In contrast to ASCs, mature adipocytes are the most abundant cell type in adipose tissue [10]. It has been shown that mature adipocytes isolated from fat tissue can be dedifferentiated into fibroblast-like cells by an in vitro dedifferentiation strategy known as ceiling culture [10]. Yagi et al. [11] has established an adipogenic progenitor cell line derived from the mature adipocytes of ddY mice and named these cells dedifferentiated fat (DFAT) cells. In addition, compared with ASCs, a relatively homogeneous cell population of DFAT cells has been revealed by flow cytometric analysis [10].

We have investigated bone tissue engineering using a combination of DFAT cells and the self-assembling peptide RADA16 or titanium fiber mesh as a scaffold and found that DFAT cells proliferate and differentiate into osteoblasts [12–14]. These findings prompted us to hypothesize that DFAT cells are multipotent cells similar to ASCs and mesenchymal stem cells. However, there are no studies comparing the osteoblastic differentiation ability of DFAT cells with ASCs.

The hypothesis more supported by the work carried out would be that DFAT cells maintain a higher degree of multipotency than ASCs, and therefore, they should have a higher osteogenic potential. Thus, the purpose of this study was to evaluate and compare the osteoblastic differentiation abilities of DFAT cells and ASCs from the BFP in vitro.

## Materials and methods

### Isolation and culture of DFAT cells and ASCs

**Fig. 1:**
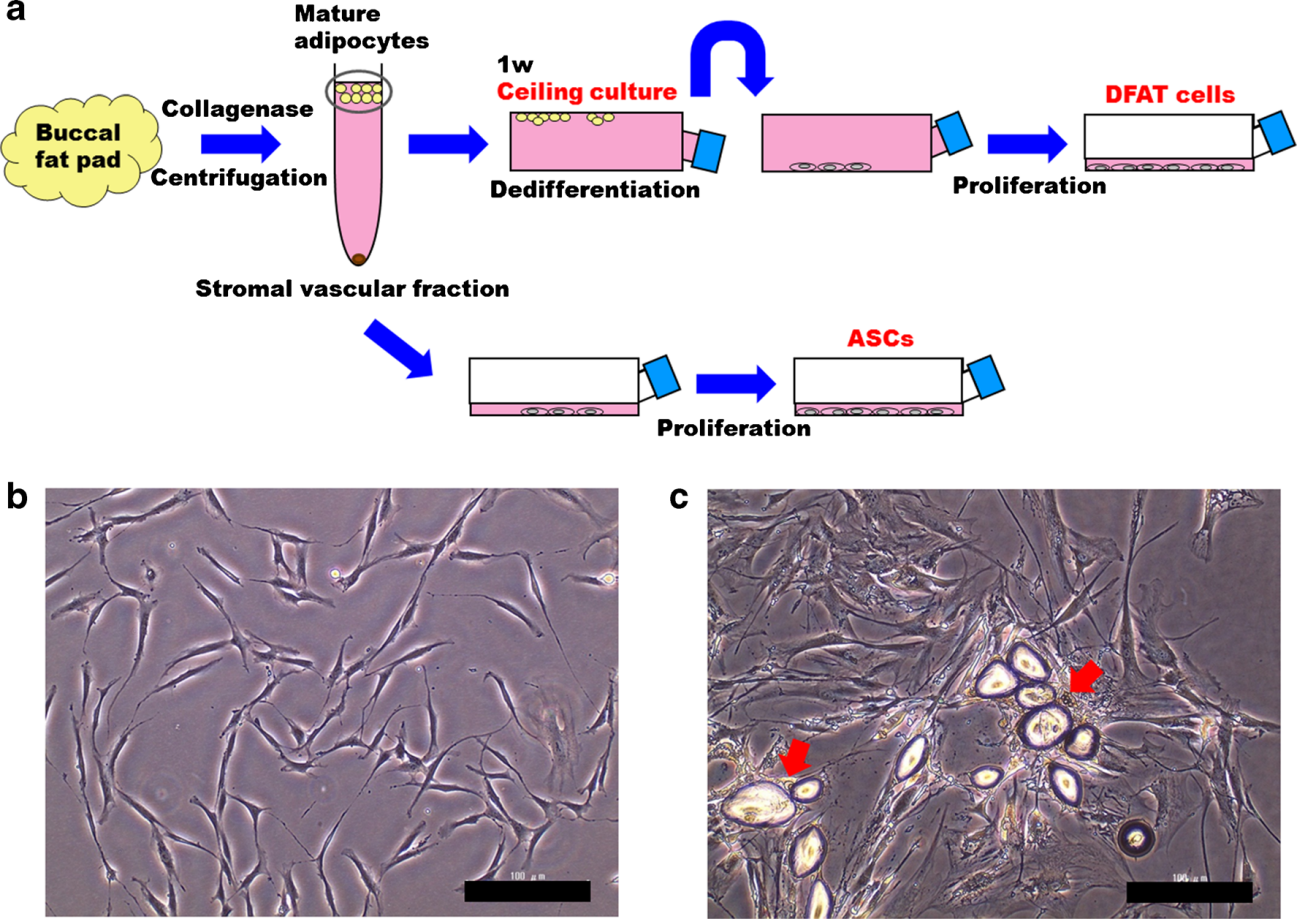
Isolation of human dedifferentiated fat (*DFAT*) cells and adipose stem cells (*ASCs*) from the buccal fat pad (**a**). Morphology of ASCs (**b**) and DFAT cells (**c**) from the BFP at 7 days after dissemination of the stromal vascular fraction and the beginning of ceiling culture, respectively. *Red arrows* indicate lipid droplets in mature adipocytes (**c**). *Scale bars* = 100 μm

A human BFP was obtained from a female patient (34 years old), who underwent oral and maxillofacial surgery at Osaka Dental University Hospital, following written informed consent. The patient was healthy and had no systemic disease. All procedures were approved by the ethics committee of Osaka Dental University (approval number 110714). We isolated DFAT cells using the “ceiling culture method” as described in our previous report [12]. Briefly, approximately 1 g of BFP was minced into small pieces and then dissociated in a 0.1 % (*w*/*v*) collagenase solution (collagenase type Ι; Wako Pure Chemical, Osaka, Japan) at 37 °C for 1 h with gentle agitation. The cell suspension was filtered through 150- and 250-μm nylon meshes to allow cells to pass through and exclude unwanted stromal cells and tissue. Floating mature adipocytes in the top layer were collected and centrifuged at 135*g* for 3 min. Isolated mature adipocytes were seeded in a 25-cm^2^ culture flask (Sumilon; Sumitomo Bakelite, Tokyo, Japan) that was completely filled with Dulbecco’s modified Eagle’s medium (DMEM; Nacalai Tesque, Kyoto, Japan) supplemented with 20 % (*v*/*v*) fetal bovine serum (FBS; lot number 1412447; Invitrogen, Life Technologies, Carlsbad, CA) and antibiotics and antimycotics (an antibiotic/antimycotic mixed stock solution consisting of 10,000 U/mL penicillin, 10,000 μg/mL streptomycin, and 25 μg/mL amphotericin B; Nacalai Tesque). The cells were then incubated at 37 °C with 5 % CO_2_. The flask was positioned with the adhesive culture surface facing upward, so that floating adipocytes containing lipid droplets attached to the inner ceiling surface of the flask. This method is referred to as “ceiling culture” (Fig. 1). After 7 days, the medium was removed and the flask was inverted. Fresh medium was added to barely cover the bottom of the flask. The medium was then changed twice a week. At confluency, the cells were passaged and used for experiments.

We isolated ASCs according to a report by Zuk et al. [1] (Fig. 1). The medium used for DFAT cell culture was also applied to ASC culture. The culture medium for both cell types was replaced twice a week. At confluency, the cells were passaged and used for experiments.

### Flow cytometric analysis

Cell surface antigens of passage 2 cells were analyzed by flow cytometry. At 90 % confluence, DFAT cells and ASCs from the BFP were detached by Accutase (Merck Millipore, Billerica, MA) and then resuspended in phosphate-buffered saline (PBS) at a density of 1 × 10^7^ cells/mL. One hundred microliters of each cell suspension was incubated with fluorescently labeled antibodies against CD11b, CD34, CD45, CD90, and CD105 (all purchased from BioLegend, San Diego, CA) in the dark for 30 min on ice. Unbound antibodies were washed out, and each cell sample was analyzed using a Guava easyCyte Flow Cytometer (Merck Millipore). Positive cells were counted and compared with the signals of the corresponding antibody isotype controls.

### DNA analysis

DFAT cells and ASCs were seeded in 12-well plates at a density of 3 × 10^4^ cells/well and incubated in standard medium at 37 °C with 5 % CO_2_. At confluency, both cell types were cultured in normal medium (NM; DMEM supplemented with 10 % FBS) or osteogenic medium [OM; NM supplemented with 10 % FBS, 100 nM dexamethasone (Sigma-Aldrich, St. Louis, MO), 50 μM l-ascorbic acid 2-phosphate (Sigma-Aldrich), and 10 mM β-glycerophosphate (Sigma-Aldrich)] for 14 days. Both medium types were replaced twice a week. DNA content was measured on days 3, 7, and 14. The medium was removed from each well, and the cell layer was washed twice with PBS. Then, 300 μL of 0.2 % Triton X-100 was added to each well, and the cells were removed using a cell scraper (BD, Franklin Lakes, NJ) to extract the DNA. DNA content was measured with a Quant-iT™ PicoGreen® dsDNA Reagent and Kit (Invitrogen). Fifty microliters of the sample was mixed with a DNA-binding fluorescent dye solution (0.5 μL PicoGreen reagent in 100 μL 1× TE buffer), and the fluorescence intensity was measured with a microplate reader (excitation 450 nm/emission 510 nm, SpectraMax® M5; Molecular Devices, Sunnyvale, CA) (*n* = 4).

### Bone-specific alkaline phosphatase measurement

Bone-specific alkaline phosphatase (BAP) activity was measured with Osteolinks BAP (DS Pharma Biomedical, Osaka, Japan). The same samples (20 μL) used for DNA measurement were added to each well of a BAP antibody-coated microtiter plate and then incubated at room temperature for 3 h. After four washes with the wash buffer supplied with the kit, 150 μL of substrate solution (disodium *p*-nitrophenyl phosphate) was added to each well, followed by incubation at room temperature for 30 min. After 100 μL of stop solution was added to each well, the absorbance was measured at 405 nm with the SpectraMax® M5 (*n* = 4).

### Osteocalcin measurement

DFAT cells and ASCs were cultured under the same condition described in “DNA analysis,” and then the expression of osteocalcin (OCN) in both cell types was determined with a Gla-type Osteocalcin EIA Kit (Takara Bio, Shiga, Japan). On days 3, 7, and 14 of culture, the medium was removed, the cells were washed with PBS, and 300 μL of 10 % formic acid was added to each well, followed by removal of the cells using a cell scraper. Samples (100 μL) were added to each well of an anti-OCN antibody-coated microtiter plate and then incubated at room temperature for 2 h. After three washes with PBS, 100 μL of a peroxidase-conjugated anti-OCN antibody solution was added to each well, followed by incubation at room temperature for 1 h. After four washes with PBS, 100 μL of substrate solution (tetramethylbenzidine) was added to each well, followed by incubation at room temperature for 15 min. After 100 μL of stop solution was added to each well, the absorbance was measured at 450 nm with the SpectraMax® M5 (*n* = 4).

### Calcium measurement

Calcium deposition was measured with a Calcium E-test Wako (Wako Pure Chemical). Two milliliters of monoethanolamine buffer was added to 50 μL of the same sample used for OCN measurement, followed by thorough mixing. After 1 mL methyl xylenol blue coloring agent was added to the mixture, the absorbance was measured at 610 nm with the SpectraMax® M5 (*n* = 4).

### Alizarin red staining

DFAT cells and ASCs were cultured under the same condition described in DNA analysis, and then, both cell types were stained using alizarin red to assess mineralization. On days 3, 7, and 14 of culture, the medium was removed, and the cells were fixed in 95 % ethanol for 10 min. Then, the cells were rinsed with distilled water and stained with 1 % alizarin red S (pH 6.1) for 30 min. The stained cells were washed with distilled water three times with gentle agitation.

### Statistical analysis

Data are expressed as the mean ± standard deviation. Statistical analysis was performed between DFAT cells and ASCs cultured in the same medium (e.g., ASC NM vs. DFAT cell NM) to compare the proliferation and osteoblastic differentiation ability of both cell types. Intergroup comparisons were performed using the unpaired *t* test. Differences were considered significant at *p* < 0.05.

## Results

### Flow cytometric analysis

**Fig. 2:**
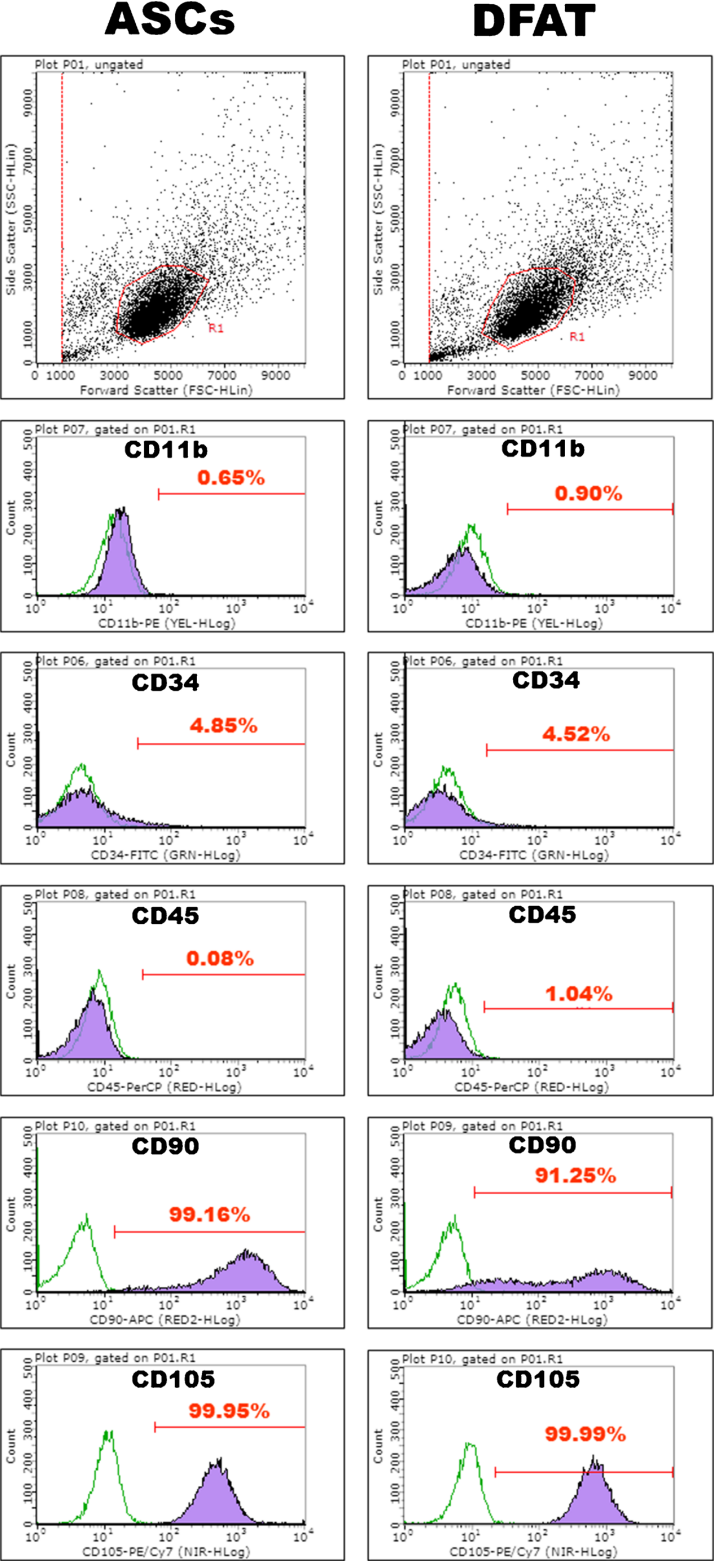
Expression of CD11b (monocyte marker), CD34 (hematopoietic progenitor cell marker), CD45 (leukocyte common antigen), and CD90 and CD105 (mesenchymal stem/progenitor cell markers) on adipose stem cells (*ASCs*) and dedifferentiated fat (*DFAT*) cells from the buccal fat pad at passage 2. *Green open histograms* represent antibody isotype controls, and *purple closed histograms* represent a sample stained with specific antibodies

Human DFAT cells from the BFP at passage 2 were positive for CD90 and CD105 (mesenchymal stem/progenitor cell markers) and negative for CD11b (monocyte marker), CD34 (hematopoietic progenitor cell marker), and CD45 (leukocyte common antigen). Similar to DFAT cells, ASCs from the BFP at passage 2 were positive for CD90 and CD105 and negative for CD11b, CD34, and CD45 (Fig. 2).

### Proliferation and osteoblastic differentiation of DFAT cells and ASCs

**Fig. 3:**
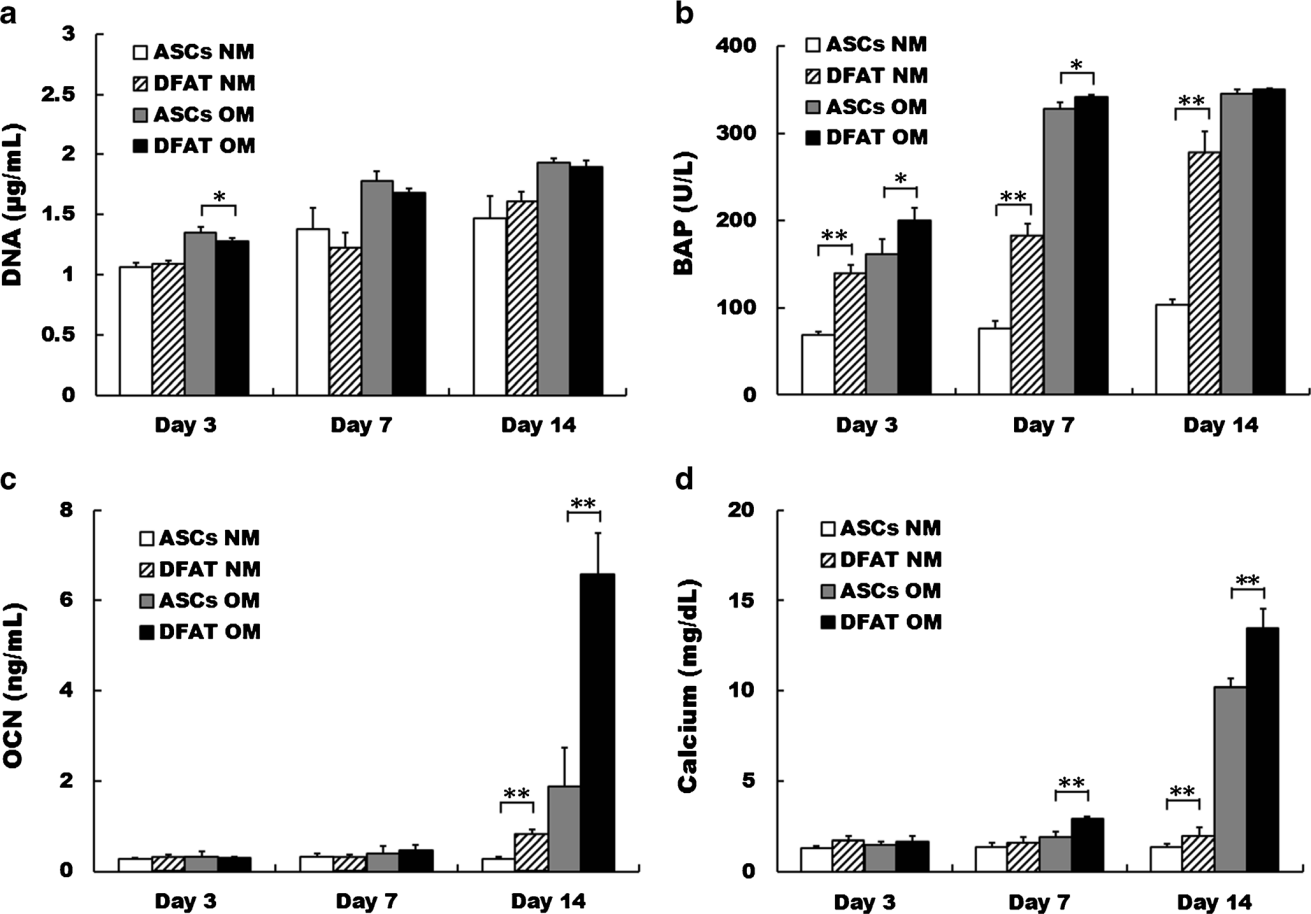
Proliferation and osteoblastic differentiation of dedifferentiated fat (*DFAT*) cells and adipose stem cells (*ASCs*). DNA (**a**), BAP (**b**), OCN (**c**), and calcium (**d**) deposition of DFAT cells and ASCs cultured in normal medium (*NM*) and osteogenic medium (*OM*). Data are the mean ± standard deviation. *p < 0.05; **p < 0.01

As an indicator of cell proliferation, the DNA content of ASCs cultured in OM was significantly higher than that of DFAT cells on day 3 (*p* < 0.05). However, no significant difference was found at all other time points. BAP is synthesized by osteoblasts and can be used as an index for the rate of bone formation [15]. The BAP expression of DFAT cells cultured in NM was significantly higher than that of ASCs on days 3, 7, and 14 (*p* < 0.01). Furthermore, BAP expression of DFAT cells cultured in OM was significantly higher than that of ASCs on days 3 and 7 (*p* < 0.05). The expression of OCN, a marker of late-stage osteoblastic differentiation [16], in DFAT cells cultured in NM and OM was significantly higher than that in ASCs on day 14 (*p* < 0.01). The calcium deposition, a marker of terminal-stage osteoblastic differentiation [17], of DFAT cells cultured in NM was significantly higher than that of ASCs on day 14 (*p* < 0.01). Moreover, the calcium deposition of DFAT cells cultured in OM was significantly higher than that of ASCs on days 7 and 14 (*p* < 0.01) (Fig. 3).

### Morphology and alizarin red staining of DFAT cells and ASCs cultured in NM and OM

**Fig. 4:**
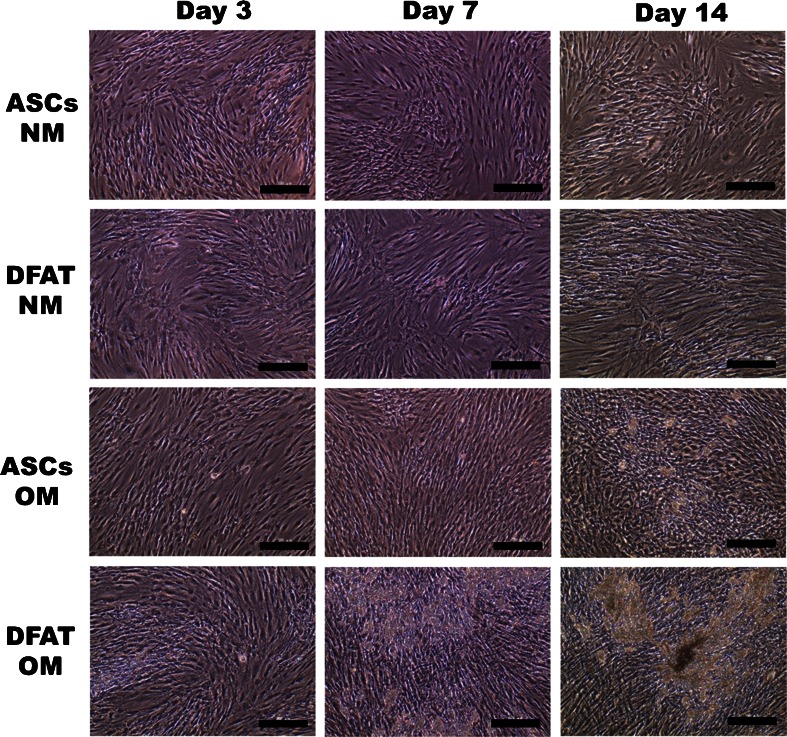
Morphology of dedifferentiated fat (*DFAT*) cells and adipose stem cells (*ASCs*) cultured in normal medium (*NM*) and osteogenic medium (OM). A *yellowish brown* mineralized extracellular matrix was slightly observed in DFAT cells cultured in OM for 7 days and in ASCs cultured in OM for 14 days. A significant mineralized extracellular matrix was observed in DFAT cells cultured in OM for 14 days. *Scale bars* = 100 μm

Under a phase contrast microscope, some mineralized nodule formations were observed in DFAT cells cultured in OM on day 7, which increased on day 14. ASCs cultured in OM showed some mineralized nodule formations on day 14 only, but they were fewer than those observed in DFAT cells. There were no mineralized nodule formations in both cell types cultured in NM at all time points (Fig. 4).

**Fig. 5:**
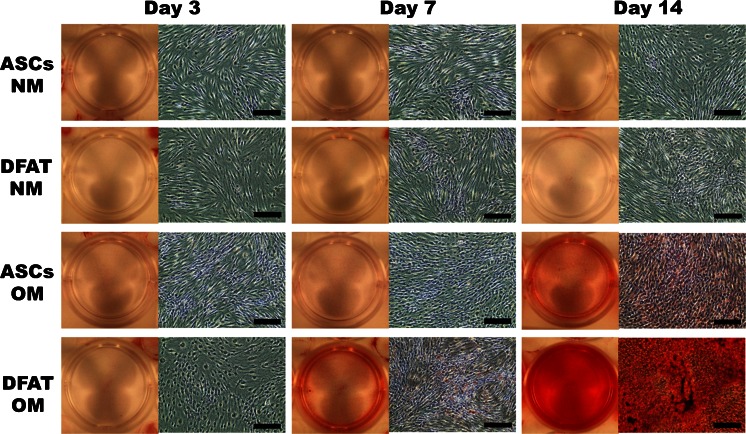
Alizarin red staining of dedifferentiated fat (*DFAT*) cells and adipose stem cells (*ASCs*) cultured in normal medium (*NM*) and osteogenic medium (*OM*). *Red areas* stained by alizarin red indicate calcium deposition. The wells containing DFAT cells cultured in OM for 7 days and ASCs cultured in OM for 14 days were stained weakly by alizarin red, whereas those containing DFAT cells cultured in OM for 14 days were stained strongly by alizarin red. *Scale bars* = 100 μm

Red areas stained by alizarin red are an indication of calcium deposition. The plates containing DFAT cells cultured in OM for 7 days and ASCs cultured in OM for 14 days were stained weakly by alizarin red. However, the plate containing DFAT cells cultured in OM for 14 days was stained strongly using alizarin red (Fig. 5).

## Discussion

In the present study, we compared the proliferation and osteoblastic differentiation of DFAT cells and ASCs. There were no significant differences in the DNA analysis of DFAT cells and ASCs, except for those cultured in OM on day 3. Therefore, the proliferation rates of DFAT cells and ASCs are considered to be similar. However, the expression of osteoblastic differentiation markers (BAP, OCN, and calcium) in DFAT cells was more prevalent than that in ASCs. These results and those of alizarin red staining indicate that the osteoblastic differentiation ability of human DFAT cells is higher than that of ASCs. Therefore, DFAT cells have an advantage over ASCs in bone tissue engineering because the induction of osteoblasts from DFAT cells is more effective.

The OM applied in this study has been widely used to induce osteoblastic differentiation of ASCs [1, 4, 18, 19] and DFAT cells [10, 12]. Therefore, we consider that the OM did not contribute to the significant differences in the expression of osteoblastic differentiation markers between ASCs and DFAT cells.

DFAT cells are isolated from mature adipocytes in adipose tissue as shown in Fig. 1. After the adipose tissue was minced and digested in a collagenase solution, the mature adipocytes containing lipid droplets were suspended and could be separated from the SVF by centrifugation. Almost all floating cells (over 98 %) have been reported to be mature adipocytes that comprise a highly homogeneous fraction [20]. Accordingly, DFAT cells have been reported as a homogeneous cell population because these cells are isolated by collecting the floating pure cell population, followed by ceiling culture using the buoyancy of the adipocytes [10, 12, 20, 21]. Conversely, ASCs are a heterogeneous cell population because they are isolated from the SVF that contains many cell types, but excluding mature adipocytes. Compared with ASCs, Matsumoto et al. have reported that DFAT cells are a much more homogeneous cell population, because human ASCs at passage 1 are 13.3 % positive for CD11b (monocyte marker) and 12.8 % positive for CD45 (leukocyte common antigen), whereas human DFAT cells at passage 1 are negative for these markers [10]. Therefore, populations of DFAT cells may include significantly more undifferentiated multipotent cells than those of ASCs. Consequently, DFAT cells might maintain a higher degree of multipotency than that of ASCs, and the osteoblastic differentiation ability of DFAT cells may be higher than that of ASCs. However, the ASCs and DFAT cells used in this study were negative for CD11b and CD45, and we did not show that the DFAT cells were a more homogeneous cell population than the ASCs population. Matsumoto et al. used ASCs and DFAT cells at passage 1 [10], whereas both cell types were used at passage 2 in this study. It is possible that the difference between our flow cytometry data and previous report data [10] was because of the difference in passage number. The difference of the osteoblastic differentiation ability of DFAT cells and ASCs was not because of the difference of purity in the cell populations. Therefore, it is unknown why the osteoblastic differentiation ability of DFAT cells is higher than that of ASCs. More recently, we have reported the difference of the osteoblastic differentiation ability of human DFAT cells and human bone marrow mesenchymal stem cells (BMSCs) [22]. Gene expression of Runx2 (a master regulator of osteoblast development), alkaline phosphatase activity, OCN expression, and calcium deposition is higher in DFAT cells than that in BMSCs, although the reasons are unclear. Based on the present study, we believe that the osteoblastic differentiation ability of DFAT cells is higher than that of not only BMSCs but also ASCs.

Our study is the first report of DFAT cells isolated from the BFP that is easily accessible, exhibits rich vascularization, and is widely used in oral surgery to repair bone and periodontal defects [23–25]. Harvesting of the BFP is a simple procedure that requires a minimal incision with local anesthesia and causes minimal donor site morbidity [4]. Furthermore, compared with subcutaneous adipose tissue, there might be an esthetic advantage using the BFP because adipose tissue can be obtained from the BFP via the oral cavity without injury to the external body surface.

In summary, the advantages of DFAT cells from the BFP are as follows: (1) their osteoblastic differentiation ability is higher than that of ASCs, and (2) they are easily harvested by an oral surgeon without an esthetic impact. We consider that DFAT cells from the BFP, which possess the beneficial characteristics mentioned above, are an ideal cell source for bone tissue engineering.
